# Effect of a Home Health and Safety Intervention on Emergency Department Use in the Frail Elderly: A Prospective Observational Study

**DOI:** 10.5811/westjem.58378

**Published:** 2023-05-03

**Authors:** Sandy Bogucki, Gina Siddiqui, Ryan Carter, Joanne McGovern, James Dziura, Geliang Gan, Fangyong Li, Gina Stover, David C Cone, Carolyn Brokowski, Daniel Joseph

**Affiliations:** * Yale School of Medicine, Department of Emergency Medicine, New Haven, Connecticut; † New York City Health and Hospitals, Elmhurst Hospital Center, Department of Emergency Medicine, Queens, New York; ‡ Our Lady of Fatima Hospital, Department of Emergency Medicine, North Providence, Rhode Island; § Yale School of Medicine, Yale Center for Analytical Science, Department of Emergency Medicine and of Endocrinology, New Haven, Connecticut; || Yale School of Public Health, Yale Center for Analytical Sciences, New Haven, Connecticut; # University of Hawai’i, Honolulu, Hawaii

## Abstract

**Introduction:**

Geriatric patients are often frail and may lose independence through a variety of mechanisms including cognitive decline, reduced mobility, and falls. Our goal was to measure the effect of a multidisciplinary home health program that assessed frailty and safety and then coordinated ongoing delivery of community resources on short-term, all-cause emergency department (ED) utilization across three study arms that attempted to stratify frailty by fall risk.

**Methods:**

Subjects became eligible for this prospective observational study via one of three pathways: 1) by visiting the ED after a fall (2,757 patients); 2) by self-identifying as at risk for falling (2,787); or 3) by calling 9-1-1 for a “lift assist” after falling and being unable to get up (121). The intervention consisted of sequential home visits by a research paramedic who used standardized assessments of frailty and risk of falling (including providing home safety guidance), and a home health nurse who aligned resources to address the conditions found. Outcomes of interest were all-cause ED utilization at 30, 60, and 90 days post-intervention compared with subjects who enrolled via the same study pathway but declined the study intervention (controls).

**Results:**

Subjects in the fall-related ED visit arm were significantly less likely to have one or more subsequent ED encounters post-intervention than controls at 30 days (18.2% vs 29.2%, P<0.001); 60 days (27.5% vs 39.8%, P<0.001); and 90 days (34.6% vs 46.2%, P<0.001). In contrast, participants in the self-referral arm had no difference in ED encounters post-intervention compared to controls at 30, 60, or 90 days (P=0.30, 0.84, and 0.23, respectively). The size of the 9-1-1 call arm limited statistical power for analysis.

**Conclusion:**

A history of a fall requiring ED evaluation appeared to be a useful marker of frailty. Subjects recruited through this pathway experienced less all-cause ED utilization over subsequent months after a coordinated community intervention than without it. The participants who only self-identified as at risk for falling had lower rates of subsequent ED utilization than those recruited in the ED after a fall and did not significantly benefit from the intervention.

## INTRODUCTION

The preservation of autonomy and the ability to live independently is a major focus of geriatric medicine.^1^,^2^,^3^ Geriatric patients are often frail and vulnerable and may lose independence through a variety of mechanisms including cognitive decline, depression, functional decline and reduced mobility, and falls.^1^,^2^ Importantly, many of these risk factors are modifiable.^4^ A growing body of research surrounding geriatric falls has demonstrated that they are both prevalent, afflicting approximately 30% of community-living persons >65 years, and significant drivers of loss of independence, often triggering nursing home placement.^5^,^6^,^7^,^8^

Approximately three million individuals are treated for falls each year in emergency departments (ED).^9^ Fall-related deaths appear to be increasing, with the US Centers for Disease Control and Prevention noting a 30% increase from 2007 to 2016.^9^ Falls are also costly both to individuals and to the healthcare system, with a median cost of more than $26,000.^10^ In 2015 the total cost associated with falls in the US exceeded $50 billion.^9^ From the community and prehospital standpoint, falls also result in significant resource expenditure and call volume to emergency medical services (EMS).^11^,^12^,^13^,^14^ Individuals who fall are also likely to have repeat EMS and ED encounters.^15^,^16^

Multiple significant barriers have limited frail elders’ acceptance of home healthcare assessments and delivery. One observed barrier for high-risk populations has been patient reluctance to admit home health personnel into their homes; however, once EMS professionals have been trained in “community paramedicine” techniques, they were able to achieve patient trust and have made significant contributions to various public health aims.^17^ A second barrier is finding a reliable marker for frailty that detects individuals likely to benefit without over-enrolling patients who will not. Falls appear to be an indicator of frailty among the elderly, although target populations in the fall-prevention literature range from healthy volunteers in day centers to hospitalized patients, and it is not discernible whether differences in effectiveness of interventions derive from diversity of the study population or the interventions themselves. A final barrier is defining success without patient-valued outcomes; most studies reported recurrent falls as an endpoint, but few measured broader, all-cause morbidity or mortality.

### Importance

Providing the elderly who are frail with the ability to maintain independence and live safely at home is of paramount importance to preserving their quality of life.^18^ Multiple interventions have been attempted to target this population with varying effect. Interventions in the ED have had limited success, with few documenting improved outcomes.^19^,^20^,^21^,^22^,^23^ Primary care-based interventions have shown similar results.^24^ ^25^ Multifactorial approaches appear to be more successful.^26^ Additionally, EMS-based interventions have shown some promise.^27^,^28^,^29^,^30^,^31^,^32^ Widespread success with home safety assessment interventions in the real world, however, remains limited as they have often not been coupled with ongoing community resources and care. This study introduces a novel approach to address this significant gap in healthcare support of the frail elderly living at home.

**Population Health Research Capsule**

What do we already know about this issue?

*Elderly falls at home requiring EMS response were associated with repeat 9-1-1 calls and transport to an ED over the following 30 days*.

What was the research question?

*Can a coordinated health and safety visit by a paramedic and a home care nurse decrease all-cause ED utilization over 30–90 days?*

What was the major finding of the study?

*Among frail elderly, the intervention reduced the proportion of repeat ED visits significantly at 30, 60, and 90 days (18.2% vs 29.2%, 27.5% vs 39.8%, and 34.6% vs 46.2%, respectively, P<0.001 for all)*.

How does this improve population health?

*The complementary skills of EMS and home care nurses can enhance the health and safety of elders, reducing their reliance on emergency medical care*.

### Goals of This Investigation

We sought to measure the effect of a coordinated frailty assessment and home safety intervention by research paramedics with follow-up visits by community-based home health nurses on subsequent, all-cause ED utilization at 30, 60, and 90 days post-intervention. Ultimately, the goal was to improve the safety of enrolled subjects and enhance their ability to live independently.

## METHODS

### Study Design and Setting

The Paramedic Referrals for Increased Independence and Decreased Disability in the Elderly (PRIDE) program was a prospective observational study, conducted between March 2015–April 2018. Subjects resided in the geographic catchment area of 15 towns in south-central Connecticut.

### Selection of Participants

Study subjects were recruited into one of three enrollment populations: 1) those who were seen in the ED after falls; 2) individuals who responded to public messaging and perceived themselves to be at risk for falling; and 3) those referred by EMS agencies after they called 9-1-1 for a “lift assist” or help getting up after a non-injury fall at home. Subjects were recruited into the ED arm by research associates (RA) stationed in the ED of a large, urban, tertiary care hospital with over 100,000 ED visits per year.

On assigned schedules that generally covered day and evening shifts seven days a week, these RAs monitored patient locator boards for chief complaints suggestive of falls by seniors. When appropriate, they approached the patient and/or family, explained the study, and if eligible, invited them to participate. Following informed consent, the participants were enrolled as study subjects. Those who were interested in participating but were admitted to the hospital on that ED visit were contacted again by the RAs near the time of discharge to facilitate entry into the study.

Subjects who perceived themselves as elderly and at risk of falling, were recruited through information events and public messaging. Recruitment efforts included tables at senior centers, senior housing complexes, churches, and other venues, which were staffed by research paramedics wearing PRIDE logos who answered questions and distributed brochures. Radio spots and billboards describing the program and providing contact information were also used.

Subjects were recruited into the EMS referral arm at the time of a “lift assist” call if they or the responsible family member at the scene agreed that a study representative could subsequently call and invite them to participate in the study. If the patient consented to the follow-up call, his or her name and telephone number were forwarded by the EMS responders to study personnel. Following informed consent, all subjects who did not wish to participate in the study intervention were given the option of declining.

In all arms of the study, eligibility was restricted to adults living at home or in assisted living facilities within the geographic catchment area. Participants living in long-term care facilities were not eligible to participate. There was no explicit age requirement, but participants were primarily over 65, likely due to use of the term “elderly” in the program title. We defined the intervention group as those who agreed to participate in the intervention. Participants comprising the control group consented to have their subsequent ED utilization followed but chose not to participate in the intervention. Each participant received a $10 gift card to a local supermarket for enrolling, and a $15 gift card for completing the interventions.

### Intervention

The intervention consisted of a visit by a Connecticut-licensed paramedic serving independently of the EMS system and trained and identified to the public as a research assistant for this project. The research paramedic performed a home safety check (availability of grab bars, working smoke detectors, risks associated with throw rugs, trip hazards, etc), obtained a list of current medications, and employed standard instruments to assess degrees of frailty.^33^,^34^,^35^,^36^ The research paramedic also contacted the study subject’s primary care clinician, discussed relevant findings from the home assessment, and if the subject consented, facilitated a follow-up visit. Free transportation to the primary care office site was offered as part of the intervention. The precise screening performed, and the field-adapted Fall Risk Inventory, can be found in Appendix 1.

Following the research paramedic’s visit, there was a pre-arranged house call by a nurse from one of several participating home health agencies. The nurse reviewed the findings of the research paramedic’s assessment, performed medication reconciliation, and confirmed access to currently prescribed medications. The nurse also determined needs for durable equipment and ongoing services such as physical or occupational therapy and arranged for delivery. Research paramedics and visiting nurse staff were formally trained for the intervention, including didactic training and opportunities to ride along with their counterparts in the care team and to shadow case managers and care coordinators in the ED. Further details on the training curriculum for paramedics and nurses can be found in Appendix 2.

Finally, after the interventions were completed, a brief satisfaction survey was mailed to each participant. This survey was adapted for PRIDE from the Centers for Medicare & Medicaid Home Health Care Consumer Assessment of Healthcare Providers and Systems, which was beta-tested on an early subset of subjects representing all three enrollment populations and did not require revision before deployment.

### Measurements

Data obtained from the participants directly at the time of enrollment, during the interventions performed by the research paramedics and visiting nurses during the home visits, and participants’ responses to the post-completion satisfaction survey, were collected and maintained using REDCap electronic data capture tools hosted at Yale University.^37^ ^38^ We captured subsequent ED admissions or hospitalizations by matching multiple identifiers in REDCap with participants’ electronic health records.

We measured ED utilization that occurred 30, 60, and 90 days after enrollment in the control group, and after the home health nurse visit was completed in the intervention group. Study subjects were considered part of the control group until both visits outlined in the intervention (research paramedic and visiting nurse) were completed. For example, subjects whose study intervention was completed over 60 days following enrollment, had 30-day and 60-day data included in the control group. The date of completion of the second visit was considered day 0 for the intervention group. Any EMS use at 30 days was also measured and published separately.^13^

### Outcomes

Primary outcomes were subsequent all-cause ED utilization.

### Analysis

We conducted a generalized estimating equation (GEE) analysis using SAS analytic software 9.4 (SAS Institute, Inc, Cary, NC) to compare the proportions of participants that had at least one ED visit during the 30, 60, or 90 days following enrollment in the control group or following completion of the visits in the intervention group. The GEE was used to accommodate repeated assessments from the participants, some of whom were sequentially included in control and then intervention groups. We similarly compared data across the three enrollment populations (ie, ED-recruited, self-referred, and EMS-referred) to determine whether the intervention appeared more or less effective among these groups. We also conducted a multivariable analysis with covariate adjustment including age, gender, and insurance type.

Additional supportive analyses were performed on the ED-enrolled subject populations to evaluate sensitivity. First, to further evaluate for any effects related to having some data from the same subjects in both intervention and no-intervention groups, we removed all the data from the crossover subjects from the dataset and only those who had never received the intervention throughout the study were compared by logistic regression with those who did. Second, to address potential bias due to variable delays between the time of enrollment in the ED and the time of the intervention, we looked at our population of crossover subjects (those who had outcomes recorded both before and after the study intervention). We performed paired analysis using GEE to compare the no-intervention phase vs the intervention phase of their study participation. The crossover subjects thus served as their own controls.

We performed person-time analysis using generalized Poisson regression to further evaluate the intervention’s effect on healthcare utilization. The statistical significance was defined as *P*<0.05, two-sided.

## RESULTS

**Figure 1:**
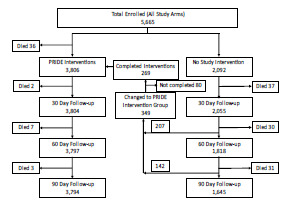
Flowchart showing total enrollment and the number of participants at each stage of the PRIDE^*^ study. The subjects who began their 30- or 60-day observation period without the PRIDE intervention but then participated in the PRIDE interventions are shown in the transition area in the middle of the chart. ^*^PRIDE, Paramedic Referrals for Increased Independence and Decreased Disability in the Elderly.

There were 5,665 individuals enrolled in the PRIDE study: 121 from 9-1-1 calls; 2,757 from ED visits; and 2,787 via self-referral. Of these, full 90-day follow-up data were available for 5,439 (96%) of enrolled subjects. Figure 1 shows the numbers of subjects and their study participation following enrollment. A few (<10) subjects contacted us requesting to withdraw from the study after initially enrolling. All of these occurred prior to an initial home visit by a PRIDE research paramedic. The records of those individuals were totally deleted from the REDCap database so that none of their personal data or medical records could subsequently be accessed by the investigators. They are not included in the total enrollment shown in the flow chart. There were 146 deaths (2.6%) of study subjects over the course of the interventions and follow-up periods; the number who died at each stage of the study is also shown in the flow chart.

Table 1 shows demographic statistics by enrollment population. The study population had an average age of 76 years, was 68% female, and 53% urban-dwelling; 81% of the participants had Medicare insurance. At least 32% of PRIDE participants lived in subsidized or public housing (data not shown), and 45% fell below the poverty line, based upon Medicaid enrollment figures. Approximately 53% of participants lived in the city of New Haven, while the rest lived in the surrounding suburbs. The self-referral arm included 73.4% participants over the age of 65 years, whereas the 9-1-1 lift assist and ED referral arms included 88.4% and 89% of subjects over 65, respectively.

Table 2 shows the main outcomes of our intervention. We found that that the PRIDE intervention had the greatest effect among those subjects invited to participate during a fall-related ED visit. In this group, the PRIDE intervention was associated with a 38% relative reduction in subsequent ED visits within 30 days, and a 25% relative reduction at 90 days of follow-up (all *P*-values significant at <0.001). The adjusted *P* value reflects demographic covariates including age, gender and insurance type(s). Individuals who entered this study through the self-referral mechanism did not have significant reductions in subsequent ED encounters (all *P*-values >0.2). Those who enrolled as a result of 9-1-1 referrals also showed no apparent benefit, although the numbers in this arm were too small for reliable comparison.

The results of the analysis for the ED-enrolled subjects excluding the intervention crossovers are presented in Table 3. The statistically significant difference between the PRIDE intervention and no intervention groups in terms of subsequent ED utilization was preserved over all three follow-up intervals with adjusted and unadjusted *P*-values <0.001.

In analyses of crossover participants only (ie, those observed during both control and intervention periods), all of the subjects had at least 30, and some up to 90 days, of outcomes data prior to receiving the intervention. As the data shown in Table 4 demonstrates, the percentage of these subjects with at least one ED visit following enrollment increased with each month of follow-up both pre- and post-intervention but was comparatively decreased following the PRIDE intervention. The differences between the groups remain statistically significant, with higher *P*-values reflecting the smaller numbers included in these subsets of study participants.

We also performed a person-time analysis to initial ED visit and an event-time analysis for all ED visits. The results are displayed in Tables 5 and 6, respectively. In the group of individuals originally recruited from the ED, the incidence rate was 3.36 per 1,000 follow-up days among intervention subjects vs 4.54 per 1,000 follow-up days in the no-intervention group, a difference that was statistically significant (*P***<0.001)**. The incidence rates of first ED visit among the 9-1-1 lift-assist and self-referral groups showed no significant difference between the intervention and no intervention groups. Incidence rates of total overall visits also demonstrated a significant difference among subjects recruited from the ED: 6.27 visits per 1,000 follow-up days in the intervention arm vs 7.16 visits per 1,000 follow-up days in the control arm (*P***<0.01**), but not among subjects recruited via 9-1-1 lift assist or self-referral.

The following question was asked as part of the participant satisfaction survey that was mailed to every subject who completed both the PRIDE paramedic and nurse visits: “Using any number from 0 to 10, where 0 is the worst healthcare experience possible and 10 is the best healthcare experience possible, what number would you use to rate your experience with the PRIDE program?” The participants were provided self-addressed, stamped envelopes for returning their surveys. A total of 3,806 surveys were mailed to participants and 1,952 were returned, for a response rate of 51%, although 77 individuals (3.9%) left this question blank. Table 7 depicts the distribution of results along the satisfaction scale described above. Of the 1,875 who answered this question, 69% rated participation in the PRIDE program a “10,” or the best healthcare experience possible.

## DISCUSSION

To our knowledge, this is the largest study of an intervention aimed at reducing short-term morbidity while maintaining independence among frail, community-dwelling older adults. Based on promising earlier studies, EMS personnel coordinated with home healthcare agency nurses and primary care physicians to address gaps in home support services and to define the individuals who were most likely to benefit from the interventions. 13

This study demonstrated decreased subsequent all-cause ED utilization at 30, 60, and 90 days after a home assessment intervention among ED patients who presented with falls, but not among individuals who self-referred. There were substantial demographic differences between the self-referred and ED-enrolled arms, as seen in Table 1. The self-referred subject group was younger, included a higher percentage of minorities, and more likely to have Medicaid or no insurance than the ED-enrolled subjects. The baseline rates of ED re-utilization in the fall-related ED visit enrollment population were two to three times the rates seen in the self-referral population. (With no intervention, 29%, 40%, and 46% of the ED subgroup visited the ED at 30, 60, and 90 days, respectively, vs 10%, 14%, and 19% of the self-referral subgroup.) This rate of subsequent ED use suggests that ED presentation by elderly individuals for falls may be a salient indicator for health systems to identify patients at high risk of returning for any reason if no intervention is performed. Based on these group comparisons, older age and falls requiring medical evaluation appeared to be more predictive of benefit from the PRIDE intervention than race or type of insurance coverage.

The significantly lower ED utilization among subjects receiving the PRIDE intervention within the ED-recruited population but not in the self-referral population further suggests that falls are a useful marker for frailty, and that the associated high risk of short-term illness and injury may be modifiable by the right set of interventions. Indeed, for patient populations not specifically restricted to falls, home visit interventions have been found to be more effective on higher risk patients.^39^ Interventions such as this are also more effective in patients who have had falls; Cumming et al and Nikolaus et al both found their interventions to be more effective in the subgroups that had previous falls.^40^,^41^

Recent research has shown that emergency physicians fail to identify risk factors for falls in the ED.^21^ Although the ED is a place where high-risk patients are concentrated, the risk mitigation strategies these patients acutely need is difficult to implement onsite, given the competing demands on a clinician’s time and the hectic environment. However, this study provides evidence that patients’ time in the ED can be harnessed effectively another way, by dedicated enrollment staff to coordinate post-visit, risk-mitigating follow-up.

In contrast, the difficulty enrolling subjects into the 9-1-1 lift-assist arm attests to the regulatory and workflow challenges for EMS personnel to enroll individuals in the same intervention. Several towns within the geographic catchment area were reluctant to allow EMS personnel to perform this enrollment, and unlike in the ED, EMS did not have additional staff helping with patient enrollment. These practical considerations are unlikely to be unique to this study and may represent reasons ED enrollment may be preferable to EMS agencies recruiting subjects on scene.

## LIMITATIONS

This was an observational cohort study that provided participants the choice whether to receive the intervention, rather than being a randomized controlled trial. Without randomization it is unknown whether selection bias is present and a contributor to the differences in outcomes between the control and intervention arms. Nevertheless, analyses of those that were observed during both control and intervention periods (ie, intervention crossovers) provided similar results. Another limitation in analysis of the intervention is that the efficacy of the PRIDE intervention was assessed in aggregate. The effect size or direction of independent components of the multifactorial intervention (research paramedic visit, nurse visit, medication reconciliation, mobility screening, primary care clinician communication, free transportation to follow-up appointments, ongoing visiting nurse services, medical equipment, etc) could not be determined from this study.

Outcomes reporting of ED visits was limited to within the Yale New Haven Health System (YNHHS); therefore, repeat ED visits to other health systems are not reflected in our analysis. However, the vast majority (at least 85%, based on EMS data) of ED visits and 88% of the inpatient beds in the study’s catchment area are at YNHHS facilities.

## CONCLUSION

Research paramedic and visiting nurse home visits were associated with lower rates of subsequent all-cause ED utilization among subjects who presented to the ED after falls but not among subjects who self-enrolled by identifying themselves as at risk for falling, nor among subjects who contacted 9-1-1 for lift assists. These findings suggest that individuals who present to the ED after falls can efficiently be enrolled and are likely to benefit from a program involving standardized home assessment of frailty and safety by specially trained paramedics and follow-up visits by home health nurses to arrange for appropriate, ongoing medical and community resources. By targeting this vulnerable group with a focused intervention, the autonomy of these patients and their ability to live independently may be enhanced and potentially preserved.

## Supplementary Information





## Figures and Tables

**Table 1:** PRIDE* participant demographics.

Characteristics	9-1-1 Lift assist (N=121)		Emergency department (N= 2,757)		Self-referral (N=2,787)	
	Number	Percentage	Number	Percentage	Number	Percentage
Age at enrollment						
Younger than 65	14	11.6%	296	11%	743	26.6%
65–74	25	20.6%	788	28.4%	679	24.3%
75–84	44	36.3%	877	31.8%	769	27.5%
85 and older	38	31.5%	796	28.8%	596	21.3%
Gender						
Female	76	63%	1834	66.6%	1,931	69.3%
Male	45	37%	923	33.4%	856	30.7%
Race						
White	96	79%	1760	64%	1,594	57%
Black	18	15%	760	27.5%	716	25.5%
Hispanic	4	3%	209	7.5%	432	15.5%
American Indian, Alaska Native, Asian/Pacific Island American, or other	3	2%	28	1%	45	2%
Insurance*						
Medicare	69	57%	1554	56%	1,128	40%
Medicaid	4	3%	240	10%	436	16%
Medicare+ Medicaid	46	38%	809	29%	1,005	36%
Private	2	2%	97	3%	106	4%
None	0	0	57	2%	112	4%

* *PRIDE*, Paramedic Referrals for Increased Independence and Decreased Disability in the Elderly.

**Table 2:** PRIDE* study outcomes comparing the percentage of study subjects with at least one subsequent, any-cause ED visit 30, 60, and 90 days following completion of the PRIDE intervention or enrollment only “No Intervention” in subjects in the 9-1-1 lift assist, self-referral, or ED enrollment populations.

Percentage of subjects with ≥1 subsequent ED encounter				

Follow-up time	No intervention	PRIDE intervention	Unadjusted P-value	Adjusted P-value
9-1-1 lift assist				
30 days	3/9 (33.33%)	22/83 (26.51%)	0.50	-
60 days	4/8 (50.00%)	28/81(34.57%)	0.38	0.36
90 days	2/6 (33.33%)	35/80(43.75%)	0.60	0.30
Self-referral				
30 days	54/516 (10.47%)	198/2,297 (8.62%)	0.31	0.30
60 days	62/440 (14.09%)	342/2,297 (14.89%)	0.56	0.84
90 days	75/393 (19.08%)	431/2,297 (18.76%)	0.66	0.23
Emergency department enrollment				
30 days	447/1,530 (29.22%)	259/1,424 (18.19%)	<0.001	<0.001
60 days	545/1,370 (39.78%)	390/1,419 (27.48%)	<0.001	<0.001
90 days	576/1,246(46.23%)	491/1,417 (34.65%)	<0.001	<0.001

* *PRIDE*, Paramedic Referrals for Increased Independence and Decreased Disability in the Elderly; *ED*, emergency department.

**Table 3:** PRIDE* study outcomes comparing the percentage of study subjects who had been enrolled in the ED and had at least one subsequent, any-cause ED visit 30, 60, and 90 days following completion of the PRIDE intervention vs enrollment only. The crossover patients included in Table 2 who had results in both the no-intervention (by virtue of time passed between enrollment and completion of the intervention) and the intervention groups were excluded in this analysis. Thus, there is no overlap between the control and intervention groups.

Percentage of ED-enrolled unique subjects with ≥1 subsequent ED encounter				
Follow-up time	No intervention	PRIDE intervention	Unadjusted P-value	Adjusted P-value
30 days	341/1,156 (29.5%)	259/1,424 (18.19%)	<0.001	<0.001
60 days	438/1,114 (39.32%)	390/1,419 (27.48%)	<0.001	<0.001
90 days	481/1,075 (45.57%)	491/1,417 (34.65%)	<0.001	<0.001

* *PRIDE*, Paramedic Referrals for Increased Independence and Decreased Disability in the Elderly; *ED*, emergency department.

**Table 4:** PRIDE* study outcomes comparing the percentage of study subjects who had been enrolled in the ED and had at least one subsequent, any-cause ED visit 30, 60, and 90 days prior to (no Intervention) or following completion of the PRIDE intervention.

Percentage of crossover ED-enrolled subjects with ≥1 subsequent ED encounter				
No intervention	PRIDE intervention	Unadjusted P-value	Adjusted	P-value
30 days	106/374 (28.34%)	74/374 (19.8%)	0.003	0.002
60 days	107/256 (41.8%)	83/256 (32.42%)	0.01	0.01
90 days	85/171 (49.7%)	69/171 (40.35%)	0.04	0.05

* *PRIDE*, Paramedic Referrals for Increased Independence and Decreased Disability in the Elderly; *ED*, emergency department.

**Table 5:** Person-time analysis for first healthcare encounter with or without PRIDE* intervention, by enrollment population.

Enrollment population	No intervention			PRIDE intervention			
	Follow-up days	# of people	# of people per 1,000 follow-up days	Follow-up days	# of people	# of people per 1,000 follow-up days	P-value
9-1-1 Lift assist	1,670	6	3.59	19,146	64	3.34	0.91
Self-referral	142,352	203	1.43	695,166	1,974	1.54	0.36
ED visits	254,595	1,156	4.54	292,016	982	3.36	<0.0001
Total overall	398,617	1,365	3.42	1,006,328	2120	2.11	<0.0001

* *PRIDE*, Paramedic Referrals for Increased Independence and Decreased Disability in the Elderly; *ED*, emergency department.

**Table 6:** Event-time analysis for all ED visits, with or without PRIDE* intervention, by enrollment population.

Enrollment population	No intervention			PRIDE intervention			
	Follow-up Days	# ED visits	# ED visits per 1,000 follow-up days	Follow-up days	# ED visits	# ED visits per 1,000 follow-up days	P-value
9-1-1 Lift assist	3,084	11	3.57	52,753	299	5.67	0.30
Self-referral	200,107	537	2.68	1,038,001	3,327	3.21	0.044
ED visits	606,716	4,343	7.16	606,578	3,801	6.27	0.0092
Total overall	809,907	4,891	6.04	1,697,332	7,427	4.38	<0.0001

* *PRIDE*, Paramedic Referrals for Increased Independence and Decreased Disability in the Elderly; *ED*, emergency department.

**Table 7:** Results of a satisfaction rating question that was part of a survey mailed to study participants after they completed both elements of the PRIDE* intervention. (See text for the wording of the question and the scale used.)

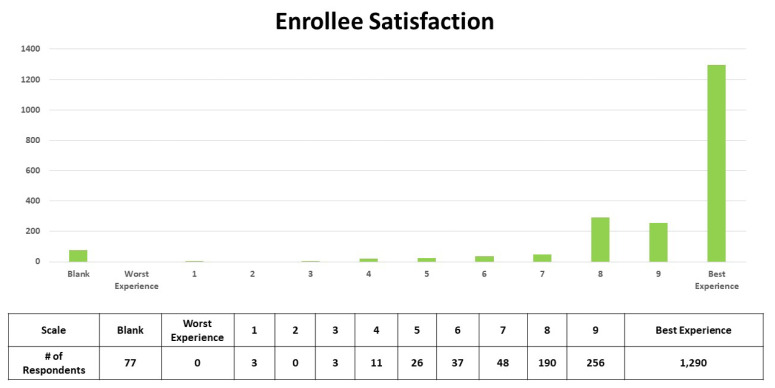

* *PRIDE*, Paramedic Referrals for Increased Independence and Decreased Disability in the Elderly.
