# Supplementary material for: Effect of a Home Health and Safety Intervention on Emergency Department Use in the Frail Elderly: A Prospective Observational Study
Source: West J Emerg Med. 2023 May 3;24(3):522–31. doi: 10.5811/westjem.58378 (PMC10284516; doi:10.5811/westjem.58378)
Supplement: Supplementary file 1 [file wjem-24-522-s001.pdf]

Demographics

Study ID ______________________________

PRIDE
PARAMEDIC REFERRALS FOR INCREASED INDEPENDENCE AND DECREASED DISABILITY IN THE ELDERLY

How were you referred to PRIDE?
- ◯ 911 Lift Assist
- ◯ Self Referral
- ◯ Emergency Department Referral

Select type of identifier
- ◯ Social Security Number
- ◯ Medicare Number
- ◯ Medicaid Number
- ◯ NA

((SSN, Medicare, Medicaid))

Identifier Number ______________________________

CONTACT/DEMOGRAPHIC INFORMATION

Today's date ______________________________
(MM/DD/YYYY)

Date subject signed consent ______________________________
(date MM/DD/YYYY)

Last name ______________________________

First name ______________________________

Address ______________________________

Town ______________________________

State ______________________________

Zip code ______________________________

Home phone ______________________________
(Example: (203) 999-9999)

Email ______________________________

Date of birth ______________________________
(date MM/DD/YYYY)

Age ______________________________
(Will automaticallly calculate)

Sex
- ◯ Female
- ◯ Male

Are you Hispanic/Latino?
- ◯ Yes
- ◯ No

Race/Ethnicity
- ☐ White
- ☐ Black
- ☐ Hispanic
- ☐ Asian/Pacific Islander
- ☐ American Indian/Alaska
- ☐ Other

Does subject live alone?
- ◯ Yes
- ◯ No

Does subject have a caregiver?
- ◯ Yes
- ◯ No

Name of caregiver ________________________________

Type of insurance
- ☐ NONE
- ☐ MEDICARE
- ☐ MEDICAID
- ☐ TRICARE
- ☐ CHAMPUS
- ☐ VA
- ☐ PRIVATE
- ☐ Medicare Advantage

PRIMARY CARE PHYSICIAN

Does subject have a Primary Physician?
- ◯ Yes
- ◯ No

If no, where does the subject receive primary care? ________________________________

Name of primary physician ________________________________

Address ________________________________

Town ________________________________

State ________________________________

Zip code ________________________________

Phone number ________________________________
(Example: (203) 999-9999)

SPECIALIST PHYSICIAN

Does subject have a specialist?
- ◯ Yes
- ◯ No

Special physician (type) ________________________________

Name of specialist ________________________________

Address ________________________________

Town ________________________________

State ______________________

Zip code ______________________

Phone number ______________________

DO NOT FORGET TO SELECT FORM STATUS AND SAVE FORM

Physical Disability

PRIDE
PARAMEDIC REFERRALS FOR INCREASED INDEPENDENCE AND DECREASED DISABILITY IN THE ELDERLY

PHYSICAL DISABILITY

Before your fall, did you need any help with everyday activities?
- ◯ Often
- ◯ Sometimes
- ◯ Only occassionally
- ◯ Rarely or Never

Score for question 1 ______________________
(Automatically calculated)

Are you able to take care of all your personal needs by yourself? (e.g., brushing your teeth, combing your hair)
- ◯ Yes (maybe with cane, walker, or other device)
- ◯ Somebody helps with these activities
- ◯ Dependent on somebody to perform these activities

Are you able to take a bath or shower by yourself?
- ◯ Yes (maybe with a cane, walker, or other device)
- ◯ Somebody helps with these activities
- ◯ Dependent on somebody to perform these activities

Are you able to dress yourself?
- ◯ Yes (maybe with a cane, walker, or other device)
- ◯ Somebody helps with these activities
- ◯ Dependent on somebody to perform these activities

Are you able to get on and off the toilet or bedside commode by yourself?
- ◯ Yes (maybe with a cane, walker, or other device)
- ◯ Somebody helps with these activities
- ◯ Dependent on somebody to perform this

Are you able to get off the bed or out of a chair by yourself?
- ◯ Yes (maybe with a cane, walker, or other device)
- ◯ Somebody helps with these activities
- ◯ Dependent on somebody to perform these activities

Are you able to walk ok, once in a standing position?
- ◯ Yes (maybe with a cane, walker, or other device)
- ◯ Somebody helps with these activities
- ◯ Dependent on somebody to perform this

Physical disability is scored automatically. Verify Physical Disability Score with table below.

SUM		DISABILITIY SCORE
16-23	=	1
13-15	=	2
10-12	=	3
7-9	=	4
6	=	5

Sum of questions ______________________

Physical Disability Score ______________________

DO NOT FORGET TO SELECT FORM STATUS AND SAVE FORM

Loss

PRIDE
PARAMEDIC REFERRALS FOR INCREASED INDEPENDENCE AND DECREASED DISABILITY IN THE ELDERLY

LOSS

In the past year, have you experienced the death or incapacity of someone who used to help take care of you? (i.e., they are now no longer able to help care for you)
- ◯ Yes
- ◯ No

If yes, who?
- ◯ Spouse/Partner
- ◯ Family Member/Relative
- ◯ Close Friend
- ◯ Paid Caregiver
- ◯ Other

If other, who? ______________________________

Is there anyone who you help care for that has suffered a serious illness or incapacity, in the past year? (i.e., are you now responsible for taking care of someone else?)
- ◯ Yes
- ◯ No

If yes, who?
- ◯ Spouse/Partner
- ◯ Family Member/Relative
- ◯ Close Friend
- ◯ Other

If other, who? ______________________________

DO NOT FORGET TO SELECT FORM STATUS AND SAVE FORM

Health Care Utilization

PRIDE
PARAMEDIC REFERRALS FOR INCREASED INDEPENDENCE AND DECREASED DISABILITY IN THE ELDERLY

HEALTH CARE UTILIZATION

How many times has someone called 9-1-1 for medical assistance for you in the past month?

- ◯ Never
- ◯ Once
- ◯ Twice
- ◯ More than twice

How many times have you been a patient in the ER in the past year?

- ◯ Never
- ◯ Once
- ◯ Twice
- ◯ More than twice

How many times have you been hospitalized (overnight) in the past year?

- ◯ Never
- ◯ Once
- ◯ Twice
- ◯ More than twice

How many times have you been admitted to an extended care facility in the past year? (i.e., assisted living, rehab, nursing home)

- ◯ Never
- ◯ Once
- ◯ Twice
- ◯ More than twice

DO NOT FORGET TO SELECT FORM STATUS AND SAVE FORM

Mental Status

PRIDE
PARAMEDIC REFERRALS FOR INCREASED INDEPENDENCE AND DECREASED DISABILITY IN THE ELDERLY

MINI-MENTAL STATUS EXAM - PART 1

Begin a mini-mental status exam. Say the following to the subject:

"I am going to name three objects, and I want you to try to remember them for me. I'll ask you to name them in a few minutes. The three objects are an egg, a boat, and a pencil."

Have the patient repeat the three items back.

INITIAL SCORE: 0 1 2 3

Give three points if the patient can repeat them back right away, two points if you have to repeat the items once, one point if you have to repeat them twice, and zero points if you have to repeat them more than twice, or if the patient just can't remember them.

Say the items again as needed until the patient can repeat all three.

DO NOT PROMPT THE SUBJECT WITH THE ITEMS AGAIN UNTIL THE SCORE HAS BEEN ASSIGNED

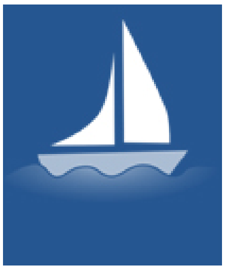
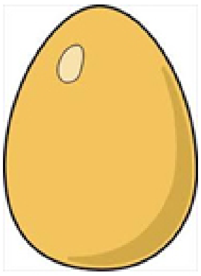
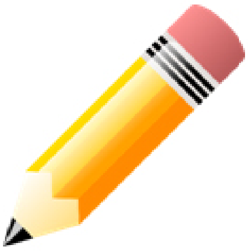

Initial Score:
◯ 0
◯ 1
◯ 2
◯ 3

Is the individual oriented to person?
◯ Yes
◯ No

Is the individual oriented to place?
◯ Yes
◯ No

Is the individual oriented to time?
◯ Yes
◯ No

Is the individual oriented to situation?
◯ Yes
◯ No

DO NOT FORGET TO SELECT FORM STATUS AND SAVE FORM

Blood Pressure

PRIDE
PARAMEDIC REFERRALS FOR INCREASED INDEPENDENCE AND DECREASED DISABILITY IN THE ELDERLY

ORTHOSTATIC BLOOD PRESSURE

1. Have the patient lie down for 5 minutes
2. Measure blood pressure and pulse rate.
3. Have the patient stand.
4. Repeat blood pressure and pulse rate measurements after standing 1 and 3 minutes.

A drop in BP of >= 20 mm Hg, or in diastolic BP of > = mm Hg, or experiencing lightheadedness or dizziness is considered abnormal.

Lying Down Systolic BP - 5 Minutes ________________________________

Lying Down Diastolic BP - 5 Minutes ________________________________

Lying Down Heart Rate - 5 Minutes ________________________________

Associated Symptoms - 5 minutes ________________________________

Standing Systolic Bp - 1 Minute ________________________________

Standing Diastolic BP - 1 Minute ________________________________

Standing Heart Rate- 1 Minute ________________________________

Associated Symptoms - 1 Minute ________________________________

Standing Systolic BP - 3 Minutes ________________________________

Standing Diastolic BP - 3 Minutes ________________________________

Standing Heart Rate- 3 Minutes ________________________________

Associated Symptoms - 3 Minutes ________________________________

DO NOT FORGET TO SELECT FORM STATUS AND SAVE FORM

Mental Status2

PRIDE
PARAMEDIC REFERRALS FOR INCREASED INDEPENDENCE AND DECREASED DISABILITY IN THE ELDERLY

MINI-MENTAL STATUS EXAM - PART 2

Once you have completed measuring orthostatic blood pressure ask the subject again:

"Can you tell me the three items I asked you to remember earlier?"

Have the patient repeat the three items back.

INITIAL SCORE: 0 1 2 3

Give three points if the patient can repeat them back right away, two points if you have to repeat the items once, one point if you have to repeat them twice, and zero points if you have to repeat them more than twice, or if the patient just can't remember them.

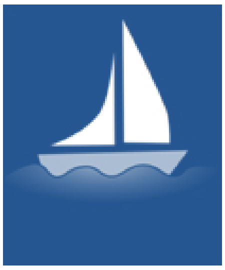

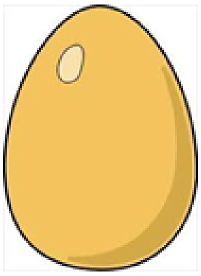

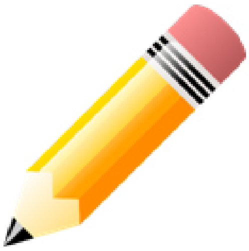

FINAL SCORE:

◯ 0
◯ 1
◯ 2
◯ 3

DO NOT FORGET TO SELECT FORM STATUS AND SAVE FORM

Risk Inventory

PRIDE
PARAMEDIC REFERRALS FOR INCREASED INDEPENDENCE AND DECREASED DISABILITY IN THE ELDERLY

FALLING - RISK INVENTORY QUESTIONNAIRE

Is it an effort for you to go down stairs?	◯ Yes<br>◯ No
Do your hands shake?	◯ Yes<br>◯ No
Do you wear shoes with rubber soles?	◯ Yes<br>◯ No
Do you use diuretics?	◯ Yes<br>◯ No
Do you get lightheaded when you urinate or have a bowel movement?	◯ Yes<br>◯ No
When you get up in the morning, do you need to remain seated on the bed for a few minutes before standing up?	◯ Yes<br>◯ No
Do you get dizzy when you turn your head?	◯ Yes<br>◯ No
Do you have trouble looking down when you walk?	◯ Yes<br>◯ No
Do you spend most of your time at home seated?	◯ Yes<br>◯ No
Do you feel faint when you stand for a while?	◯ Yes<br>◯ No
In the morning, do you need to sit up and stand up slowly?	◯ Yes<br>◯ No
Do you experience problems lacing your shoes?	◯ Yes<br>◯ No
Do you experience problems in getting dressed while standing?	◯ Yes<br>◯ No
When you walk, do your legs hurt?	◯ Yes<br>◯ No
When you are at home, do you wear slippers?	◯ Yes<br>◯ No

DO NOT FORGET TO SELECT FORM STATUS AND SAVE FORM

Assistance Needs

PRIDE
PARAMEDIC REFERRALS FOR INCREASED INDEPENDENCE AND DECREASED DISABILITY IN THE ELDERLY

ASSISTANCE NEEDS

Do you have people coming to your home to assist you?	◯ Yes<br>◯ No
Are you comfortable with having people coming into your home to assist you?	◯ Yes<br>◯ No
Do you drive?	◯ Yes<br>◯ No
Are you familiar with the resources that are available to you in the community?	◯ Yes<br>◯ No
Do you use public transportation?	◯ Yes<br>◯ No
Do you have someone drive you to your doctor's appointments?	◯ Yes<br>◯ No
Do you want to stay in your home as long as possible?	◯ Yes<br>◯ No
Do you worry about being able to maintain your independence?	◯ Yes<br>◯ No

DO NOT FORGET TO SELECT FORM STATUS AND SAVE FORM

Home Safety

PRIDE
PARAMEDIC REFERRALS FOR INCREASED INDEPENDENCE AND DECREASED DISABILITY IN THE ELDERLY

HOME SAFETY CHECKLIST

PORCH, YARD, OUTSIDE

Is the path from the house to the garage well lit?
◯ Yes
◯ No
◯ NA

Are there cracks or buckles on the sidewalks or driveway?
◯ Yes
◯ No
◯ NA

Are there hoses, weeds or other obstacles on the walkways?
◯ Yes
◯ No

Are your steps or walkways icy steps in the winter?
◯ Yes
◯ No

BATHROOM

Is the path from the bedroom to the bathroom well lit?
◯ Yes
◯ No

Are there grab bars near the toilet and in the shower and bathtub?
◯ Yes
◯ No

Does the shower have a shower seat?
◯ Yes
◯ No

Do the bathmats have slip-resistant backing?
◯ Yes
◯ No

Is there soap buildup in the shower/bathtub?
◯ Yes
◯ No

Can the subject reach soap in the shower without bending down or turning too far around (more than 90 degrees)?
◯ Yes
◯ No

Does the subject have a standard (15 inch high) toilet seat?
◯ Yes
◯ No

Does the subject have an ADA (17-19 inch high) toilet seat?
◯ Yes
◯ No

BEDROOM

Is there a table close to your bed with a lamp and room to store eyeglasses and a phone?
◯ Yes
◯ No

Are cords pushed back against the wall?
◯ Yes
◯ No

Is there clutter on the floor?
◯ Yes
◯ No

KITCHEN

Are throw rugs/floor mats secure?
◯ Yes
◯ No

Can you get to regularly used items without bending down or reaching up too far (beyond flat footed reach)?
◯ Yes
◯ No

Does the home have a working smoke detector?
◯ Yes
◯ No

LIVING AREA

Are floor coverings secure and sturdy?
◯ Yes
◯ No

Can the subject answer the phone without getting up?
◯ Yes
◯ No

Can the subject turn on a light without having to walk into a dark room?
◯ Yes
◯ No

Does the subject have a cordless or cellular phone or an emergency alarm device?
◯ Yes
◯ No

Is the floor free of clutter?
◯ Yes
◯ No

Is it easy to walk around the furniture in the home?
◯ Yes
◯ No

Can the subject pull cords to lights or ceiling fans without reaching up?
◯ Yes
◯ No

Are there handrails on both sides of the stairways in the home?
◯ Yes
◯ No

Are the steps on the stairways even and safe?
◯ Yes
◯ No

Is there adequate lighting in the stairwell with light switches at the top and the bottom of the stairs?
◯ Yes
◯ No

DO NOT FORGET TO SELECT FORM STATUS AND SAVE FORM

Timed Up and Go Test

PRIDE
PARAMEDIC REFERRALS FOR INCREASED INDEPENDENCE AND DECREASED DISABILITY IN THE ELDERLY

BALANCE ASSESSMENT

THE TIMED UP AND GO (TUG) TEST

PURPOSE: To assess mobility. An adult who takes > = 12 seconds to complete the TUG is at high risk for falling.

DIRECTIONS: Patients wear their regular footwear and can use a walking aid if needed.

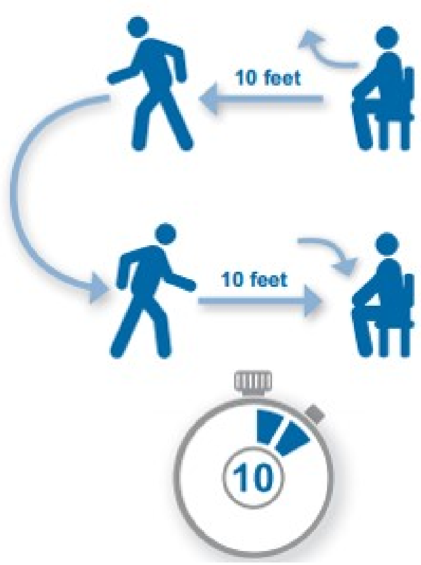

Instructions

- Ask the patient to sit in a standard chair.
- Tape a line on the floor 10 feet away.
- Tell the patient to *"Stand up from the chair, walk at your normal pace to the line on the floor, turn, walk back to the chair, and sit down again."*
- Repeat 3 times and average trials 2 and 3.
- Average time > 12 seconds suggests high risk.

In addition, the TUG test may reveal several characteristic gait patterns.

Normative Reference Values by Age

Age Group	Time in Seconds (95% Confidence Interval)	
60 – 69 years	8.1	(7.1 – 9.0)
70 – 79 years	9.2	(8.2 – 10.2)
80 – 99 years	11.3	(10.0 – 12.7)

Assess mobility using the TUG Test ______________________
(Measure in seconds)

OBSERVATION

Slow tentative pace
◯ Yes
◯ No

Loss of balance
◯ Yes
◯ No

Short strides	◯ Yes<br>◯ No
Little or no arm swing	◯ Yes<br>◯ No
Steadying self on walls	◯ Yes<br>◯ No
Shuffling	◯ Yes<br>◯ No
En bloc turning (The head's fixed to the body and joints are stiff. Turns are slow, often via multiple steps instead of a pivot)	◯ Yes<br>◯ No
Not using assistive device properly	◯ Yes<br>◯ No
Sits down in the chair hard	◯ Yes<br>◯ No
Has difficulty getting out of the chair	◯ Yes<br>◯ No

DO NOT FORGET TO SELECT FORM STATUS AND SAVE FORM

Chair Stand

PRIDE
PARAMEDIC REFERRALS FOR INCREASED INDEPENDENCE AND DECREASED DISABILITY IN THE ELDERLY

BALANCE ASSESSMENT

PURPOSE: To test leg strength and endurance. A below average score indicates a high risk for falls.

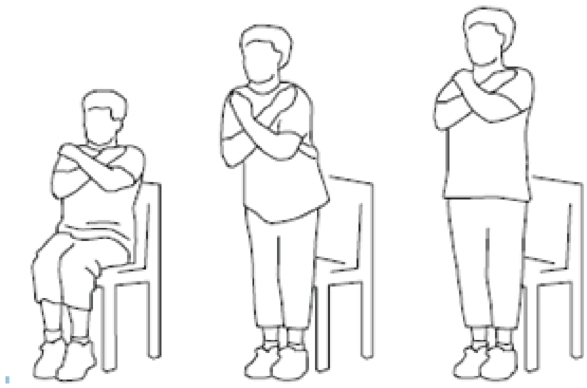

On **"Go,"** begin timing.

If the patient must use his/her arms to stand, stop the test. Record "0" for the number and score.
Count the number of times the patient comes to a full standing position in 30 seconds.
If the patient is over halfway to a standing position when 30 seconds have elapsed, count it as a stand.

Record the number of times the patient stands in 30 seconds.

CHAIR STAND—BELOW AVERAGE SCORES

Age	Men	Women
60-64	< 14	< 12
65-69	< 12	< 11
70-74	< 12	< 10
75-79	< 11	< 10
80-84	< 10	< 9
85-89	< 8	< 8
90-94	< 7	< 4

Number of times in 30 seconds subject can stand. ______________________

DO NOT FORGET TO SELECT FORM STATUS AND SAVE FORM

Functional Reach

PRIDE
PARAMEDIC REFERRALS FOR INCREASED INDEPENDENCE AND DECREASED DISABILITY IN THE ELDERLY

BALANCE ASSESSMENT

FUNCTIONAL REACH TEST

PURPOSE: A quick and simple, single-task dynamic test that defines functional reach as the maximal distance one can reach forward beyond arm's length, while maintaining a fixed base of support in the standing position.

INSTRUCTIONS:

1. Tape a line on the floor and a yardstick to the wall.

2. Explain to the participant:

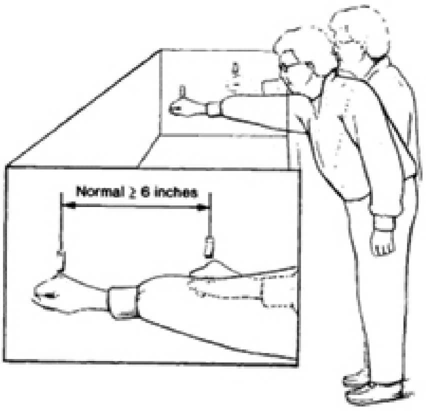

"I am interested in how far you can reach forward while you are standing. It is important that your feet stay in the same place and that you do not fall. I will ask you to stand sideways next the wall and place a ruler horizontally on the wall at your shoulder height. Then you will raise your straight arms out in front of you and make a fist. This is the starting position. I will mark this point on the ruler. Then I will ask you to reach forward without moving your feet whilst keeping your hands in a fist shape. I will then mark this new position and ask you to return to the starting position."

3. Demonstrate for the subject.

4. The subject is instructed to stand next to, but not touching the wall and to position the arm that is closer to the wall at 90° of shoulder flexion with a closed fist.

5. Place the ruler horizontally on the wall and secure appropriately. Scores are determined by assessing the difference between the start and end position is the reach distance in inches.

A Reach Score of 6 inches or less indicates that an individual is at a high risk of for falls.

Record starting position at 3rd metacarpal head on the ruler. __________________ (Measure in inches)

Ask the subject to "Reach forward as far as you can without taking a step and keeping your hands in a fist shape."

Measure location of 3rd metacarpal head on the ruler. __________________ (Measure in inches)

Second time - With the subject in the starting position - measure location of 3rd metacarpal head on the ruler. __________________ (Measure in inches)

Second time - Have the subject reach - measure location of 3rd metacarpal head on the ruler. ______________________ (Measure in inches)

What is the average distance reached? ______________________

DO NOT FORGET TO SELECT FORM STATUS AND SAVE FORM

Stage4 Balance Test

PRIDE
PARAMEDIC REFERRALS FOR INCREASED INDEPENDENCE AND DECREASED DISABILITY IN THE ELDERLY

BALANCE ASSESSMENT

THE 4-STAGE BALANCE TEST

PURPOSE: To assess static balance. An older adult who cannot hold the tandem stance for at least 10 seconds is at increased risk of falling.

DIRECTIONS:
- There are four progressively more challenging positions. Patients should not use an assistive device (cane or walker) and keep their eyes open.
- Describe and demonstrate each position. Stand next to the patient, hold his/her arm and help them assume the correct foot position.
- When the patient is steady, let go, but remain ready to catch the patient if he/she should lose their balance.
- If the patient can hold a position for 10 seconds without moving his/her feet or needing support, go on to the next position. If not, stop the test.

INSTRUCTION TO SUBJECT:
I'm going to show you four positions. Try to stand in each position for 10 seconds. You can hold your arms out or move your body to help keep your balance but don't move your feet. Hold this position until I tell you to stop.

For each stage, say "**Ready, begin**" and begin timing. After 10 seconds, say "**Stop.**"

FEET SIDE BY SIDE

Stand with feet side by side.

____
(Time in seconds)

INSTEP OF ONE FOOT TOUCHING BIG TOE OF OTHER FOOT

Place the instep of one foot so it is touching the big toe of the other foot.

____
(Time in seconds)

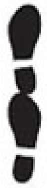

ONE FOOT IN FRONT OF THE OTHER HEEL TOUCHING TOE

Place one foot in front of the other heel touching toe. ______________________ (Time in seconds)

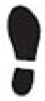

STAND ON ONE FOOT

Stand on one foot. ______________________ (Time in seconds)

DO NOT FORGET TO SELECT FORM STATUS AND SAVE FORM

Notes1

PRIDE

PARAMEDIC REFERRALS FOR INCREASED INDEPENDENCE AND DECREASED DISABILITY IN THE ELDERLY

NOTES

Notes ____________________________

DO NOT FORGET TO SELECT FORM STATUS AND SAVE FORM

Appointment Form

PRIDE
PARAMEDIC REFERRALS FOR INCREASED INDEPENDENCE AND DECREASED DISABILITY IN THE ELDERLY

REFERRAL FORM FOR RESEARCHERS

Name: [fname] [lname]

Address: [street_address]

Town/State/Zip: [town], [state], [zip]

Home Phone: [hphone]

Email: [email]

PRIMARY CARE REFERRAL

Primary Physician: [pp_name]

Address: [pp_st_address]

Town/State/Zip: [pp_town], [pp_state],[pp_zip]

Phone: [pp_phone]

Date of appointment ______________________ (MM/DD/YYYY)

Time of appointment ______________________ (HH:MM)

TRANSPORTATION ARRANGEMENTS

Select transportation service

○ Metro Taxi
○ AMR

METRO TAXI
777-7777

Time of pickup ________________ (HH:MM)

LEAVE BEHIND THE APPROPRIATE REMINDER CARD FOR THE PATIENT

VNA APPOINTMENT

Does patient have an existing VNA? ◯ Yes ◯ No

If yes, name of existing VNA. ________________

VNA - Name\Phone

(When calling specify this is a PRIDE referral.
If needed, ask for contact listed.)

Date of home visit ________________ (MM/DD/YYYY)

Time of home visit ________________ (HH:MM)

REASON FOR REFERRAL

Gait or mobility problems ◯ Yes ◯ No

Balance Difficulties ◯ Yes ◯ No

Lower Body Weakness ◯ Yes ◯ No

Medication Review & Consultation ◯ Yes ◯ No

Postural Hypotension ◯ Yes ◯ No

Other reason for follow up ________________

NOTES

Notes ________________________________

DO NOT FORGET TO SELECT FORM STATUS AND SAVE FORM

VNA Use

PRIDE
PARAMEDIC REFERRALS FOR INCREASED INDEPENDENCE AND DECREASED DISABILITY IN THE ELDERLY

REFERAL FORM FOR VNA

Name: [paramedics_arm_1][fname] [paramedics_arm_1][lname]

Address: [paramedics_arm_1][street_address]

Town/State/Zip: [paramedics_arm_1][town], [paramedics_arm_1][state], [paramedics_arm_1][zip]

Home Phone: [paramedics_arm_1][hphone]

Email: [paramedics_arm_1][email]

Date of VNA Appointment: [paramedics_arm_1][vnappoint]

Time of VNA Appointment: [paramedics_arm_1][vnatime]

PRIMARY CARE REFERAL

Primary Physician: [paramedics_arm_1][pp_name]

Address: [paramedics_arm_1][pp_st_address]

Town/State/Zip:: [paramedics_arm_1][pp_town], [paramedics_arm_1][pp_state], [paramedics_arm_1][pp_zip]

Phone: [paramedics_arm_1][pp_phone]

Date of Appointment:: [paramedics_arm_1][ppappoint]

Time of Appointment: [paramedics_arm_1][pptime]

VNA PATIENT REVIEW

Is the patient alone for more than 24 hours without someone checking on them?	◯ Yes<br>◯ No
Gait or Mobility Problems	◯ Yes<br>◯ No
Does the patient hesitate when they get up to move?	◯ Yes<br>◯ No
Balance Difficulties	◯ Yes<br>◯ No
Lower Body Weakness	◯ Yes<br>◯ No

Suspected Neurological Condition
◯ Yes
◯ No

Medication Review & Consultation
◯ Yes
◯ No

Does the patient have any dangerous medication interactions?
◯ Yes
◯ No

If yes, how many dangerous medication interactions does the patient have? ______________________

MEDICATION INTERACTION 1 - Drug 1 name ______________________

Drug 2 name ______________________

Potential effect ______________________

Recommendation ______________________

MEDICATION INTERACTION 2 - Drug 1 name ______________________

Drug 2 name ______________________

Potential effect ______________________

Recommendation ______________________

MEDICATION INTERACTION 3 - Drug 1 name ______________________

Drug 2 name ______________________

Potential effect ______________________

Recommendation ______________________

MEDICATION INTERACTION 4 - Drug 1 name ______________________

Drug 2 name ______________________

Potential effect ______________________

Recommendation ______________________

MEDICATION INTERACTION 5 - Drug 1 name ______________________

Drug 2 name ______________________

Potential effect ______________________

Recommendation ______________________

Postural Hypotension
◯ Yes
◯ No

Foot Abnormalities
◯ Yes
◯ No

Vision
☐ Glasses
☐ Impaired
☐ Blurred

Problems With Heart Rate and/or Rhythm
☐ Rate
☐ Rhythm
☐ Both

Environmental Safeguards
◯ Yes
◯ No

Do you take sleeping aids? ◯ Yes ◯ No

If yes, please list sleeping aid(s). __________________________________

Medication Change Recommended ◯ Yes ◯ No

Medication Change __________________________________

Durable Medical Equipment ◯ Yes ◯ No

List durable medical equipment __________________________________

Is the durable medical equipment in good repair? ◯ Yes ◯ No

Notes __________________________________

Do you have an Emergency Response System? ◯ Yes ◯ No

If yes, please specify EMR. __________________________________

If no, are you interested in an EMR? ◯ Yes ◯ No

ONGOING CARE NEEDS (FILLED IN BY VNA)

Physical Therapy ◯ Yes ◯ No

OCC Therapy ◯ Yes ◯ No

Home Nursing ◯ Yes ◯ No

Home Safety ◯ Yes ◯ No

Ongoing Care Needs ◯ Yes ◯ No

Does the patient present any apparent mental status problems? ◯ Yes ◯ No

Do you recommend that their primary caregiver become their primary contact? ◯ Yes ◯ No

VNA Recommendations/Notes __________________________________

DO NOT FORGET TO SELECT FORM STATUS AND SAVE FORM

Office Use - Hospital

PRIDE
PARAMEDIC REFERRALS FOR INCREASED INDEPENDENCE
AND DECREASED DISABILITY IN THE ELDERLY

HOSPITALIZATION

Has the subject been hospitalized since entering the program?
- ◯ Yes
- ◯ No

How many times?
- ◯ 1
- ◯ 2
- ◯ 3
- ◯ 4
- ◯ 5

Which hospital ______________________________

Reason for admission? ______________________________

Date of admission ______________________________
(MM/DD/YYYY)

Was surgery required?
- ◯ Yes
- ◯ No

Discharge summary admission

Date of discharge ______________________________
(MM/DD/YYYY)

Length of stay ______________________________

Cost of stay ______________________________

HOSPITAL STAY 2

Reason for second admission? ______________________________

Date of second admission ______________________________
(MM/DD/YYYY)

Was surgery required for second admission?
- ◯ Yes
- ◯ No

Discharge summary second admission

Date of second discharge ______________________________
(MM/DD/YYYY)

Length of stay second admission ______________________________

Cost of stay second admission ______________________________

HOSPITAL STAY 3

Reason for third admission? ______________________________

Date of third admission ________________________________
(MM/DD/YYYY)

Was surgery required for third admission?
◯ Yes
◯ No

Discharge summary third admission

Date of third discharge ________________________________
(MM/DD/YYYY)

Length of stay third admission ________________________________

Cost of stay third admission ________________________________

HOSPITAL STAY 4

Reason for fourth admission ________________________________

Date of fourth admission ________________________________
(MM/DD/YYYY)

Was surgery required for fourth admission?
◯ Yes
◯ No

Discharge summary fourth admission

Date of fourth discharge ________________________________
(MM/DD/YYYY)

Length of stay of fourth admission ________________________________

Cost of stay fourth admission ________________________________

HOSPITAL STAY 5

Reason for fifth admission ________________________________

Date of fifth admission ________________________________
(MM/DD/YYYY)

Was surgery required for fifth admission?
◯ Yes
◯ No

Discharge summary fifth admission

Date of fifth discharge ________________________________
(MM/DD/YYYY)

Length of stay of fifth admission ________________________________

Cost of stay fifth admission ________________________________

DO NOT FORGET TO SELECT FORM STATUS AND SAVE FORM

Office Use - Death

PRIDE
PARAMEDIC REFERRALS FOR INCREASED INDEPENDENCE AND DECREASED DISABILITY IN THE ELDERLY

Date of death ______________________
(MM/DD/YYYY)

Cause of death ______________________

DO NOT FORGET TO SELECT FORM STATUS AND SAVE FORM

Office Use - Completion Data

PRIDE
PARAMEDIC REFERRALS FOR INCREASED INDEPENDENCE AND DECREASED DISABILITY IN THE ELDERLY

STUDY INFORMATION

Subject completed study
◯ Yes
◯ No

Subject withdrawal date ______________________
(MM/DD/YYYY)

Reason for withdrawal ______________________

Study completion date ______________________
(MM/DD/YYYY)

GENERAL COMMENTS

Comments about the study ______________________

DO NOT FORGET TO SELECT FORM STATUS AND SAVE FORM

Office Use - Medication

PRIDE
PARAMEDIC REFERRALS FOR INCREASED INDEPENDENCE AND DECREASED DISABILITY IN THE ELDERLY

MEDICATION LIST

How many medications do you take? ______________________________
(Indicate number of medications up to 30.

In the past month, has there been a change to your medications?
- ◯ Yes
- ◯ No
- ◯ NA

If yes, how?
- ◯ New medication
- ◯ stopped medication
- ◯ change in dosage
- ◯ change in frequency

Which medication? ______________________________

Name of Medication 1 ______________________________

Class of Medication
- ◯ Anticoagulants
- ◯ Antidepressants
- ◯ Antipsychotics
- ◯ Beta-blockers
- ◯ Diuretics
- ◯ (Other) Antihypertensives
- ◯ NSAIDs
- ◯ Narcotics
- ◯ Opioids
- ◯ Benzodiazepines
- ◯ (Other) Sedatives
- ◯ Other

Dosage ______________________________

Frequency
- ◯ 1xday
- ◯ 2xday
- ◯ 3xday
- ◯ 4xday
- ◯ PRN

If PRN How often do you take the medication? ______________________________

Name of Medication 2 ______________________________

Class Of Medication

- ◯ Anticoagulants
- ◯ Antidepressants
- ◯ Antipsychotics
- ◯ Beta-blockers
- ◯ Diuretics
- ◯ (Other) Antihypertensives
- ◯ NSAIDs
- ◯ Narcotics
- ◯ Opioids
- ◯ Benzodiazepines
- ◯ (Other) Sedatives
- ◯ Other

Dosage ________________________________

Frequency

- ◯ 1xday
- ◯ 2xday
- ◯ 3xday
- ◯ 4xday
- ◯ PRN

If PRN How often dow you take the medication? ________________________________

Name of Medication 3 ________________________________

Class Of Medication

- ◯ Anticoagulants
- ◯ Antidepressants
- ◯ Antipsychotics
- ◯ Beta-blockers
- ◯ Diuretics
- ◯ (Other) Antihypertensives
- ◯ NSAIDs
- ◯ Narcotics
- ◯ Opioids
- ◯ Benzodiazepines
- ◯ (Other) Sedatives
- ◯

Dosage ________________________________

Frequency

- ◯ 1xday
- ◯ 2xday
- ◯ 3xday
- ◯ 4xday
- ◯ PRN

If PRN How often dow you take the medication? ________________________________

Name of Medication 4 ________________________________

Class of Medication

- ◯ Anticoagulants
- ◯ Antidepressants
- ◯ Antipsychotics
- ◯ Beta-blockers
- ◯ Diuretics
- ◯ (Other) Antihypertensives
- ◯ NSAIDs
- ◯ Narcotics
- ◯ Opioids
- ◯ Benzodiazepines
- ◯ (Other) Sedatives
- ◯

Dosage ________________________________

Frequency
- ◯ 1xday
- ◯ 2xday
- ◯ 3xday
- ◯ 4xday
- ◯ PRN

If PRN How often dow you take the medication? __________________________________

Name of Medication 5 __________________________________

Class of Medication
- ◯ Anticoagulants
- ◯ Antidepressants
- ◯ Antipsychotics
- ◯ Beta-blockers
- ◯ Diuretics
- ◯ (Other) Antihypertensives
- ◯ NSAIDs
- ◯ Narcotics
- ◯ Opioids
- ◯ Benzodiazepines
- ◯ (Other) Sedatives
- ◯

Dosage __________________________________

Frequency
- ◯ 1xday
- ◯ 2xday
- ◯ 3xday
- ◯ 4xday
- ◯ PRN

If PRN How often dow you take the medication? __________________________________

Name of Medication 6 __________________________________

Class of Medication
- ◯ Anticoagulants
- ◯ Antidepressants
- ◯ Antipsychotics
- ◯ Beta-blockers
- ◯ Diuretics
- ◯ (Other) Antihypertensives
- ◯ NSAIDs
- ◯ Narcotics
- ◯ Opioids
- ◯ Benzodiazepines
- ◯ (Other) Sedatives
- ◯

Dosage __________________________________

Frequency
- ◯ 1xday
- ◯ 2xday
- ◯ 3xday
- ◯ 4xday
- ◯ PRN

If PRN How often dow you take the medication? __________________________________

Name of Medication 7 __________________________________

Class of Medication

- ◯ Anticoagulants
- ◯ Antidepressants
- ◯ Antipsychotics
- ◯ Beta-blockers
- ◯ Diuretics
- ◯ (Other) Antihypertensives
- ◯ NSAIDs
- ◯ Narcotics
- ◯ Opioids
- ◯ Benzodiazepines
- ◯ (Other) Sedatives
- ◯

Dosage ________________________________

Frequency

- ◯ 1xday
- ◯ 2xday
- ◯ 3xday
- ◯ 4xday
- ◯ PRN

If PRN How often dow you take the medication? ________________________________

Name of Medication 8 ________________________________

Class of Medication

- ◯ Anticoagulants
- ◯ Antidepressants
- ◯ Antipsychotics
- ◯ Beta-blockers
- ◯ Diuretics
- ◯ (Other) Antihypertensives
- ◯ NSAIDs
- ◯ Narcotics
- ◯ Opioids
- ◯ Benzodiazepines
- ◯ (Other) Sedatives
- ◯

Dosage ________________________________

Frequency

- ◯ 1xday
- ◯ 2xday
- ◯ 3xday
- ◯ 4xday
- ◯ PRN

If PRN How often dow you take the medication? ________________________________

Name of Medication 9 ________________________________

Class of Medication

- ◯ Anticoagulants
- ◯ Antidepressants
- ◯ Antipsychotics
- ◯ Beta-blockers
- ◯ Diuretics
- ◯ (Other) Antihypertensives
- ◯ NSAIDs
- ◯ Narcotics
- ◯ Opioids
- ◯ Benzodiazepines
- ◯ (Other) Sedatives
- ◯

Dosage ________________________________

Frequency
- ◯ 1xday
- ◯ 2xday
- ◯ 3xday
- ◯ 4xday
- ◯ PRN

If PRN How often dow you take the medication? ______________________

Name of Medication 10 ______________________

Class of Medication
- ◯ Anticoagulants
- ◯ Antidepressants
- ◯ Antipsychotics
- ◯ Beta-blockers
- ◯ Diuretics
- ◯ (Other) Antihypertensives
- ◯ NSAIDs
- ◯ Narcotics
- ◯ Opioids
- ◯ Benzodiazepines
- ◯ (Other) Sedatives
- ◯

Dosage ______________________

Frequency
- ◯ 1xday
- ◯ 2xday
- ◯ 3xday
- ◯ 4xday
- ◯ PRN

If PRN How often dow you take the medication? ______________________

Name of Medication 11 ______________________

Class of Medication
- ◯ Anticoagulants
- ◯ Antidepressants
- ◯ Antipsychotics
- ◯ Beta-blockers
- ◯ Diuretics
- ◯ (Other) Antihypertensives
- ◯ NSAIDs
- ◯ Narcotics
- ◯ Opioids
- ◯ Benzodiazepines
- ◯ (Other) Sedatives
- ◯

Dosage ______________________

Frequency
- ◯ 1xday
- ◯ 2xday
- ◯ 3xday
- ◯ 4xday
- ◯ PRN

If PRN How often dow you take the medication? ______________________

Name of Medication 12 ______________________

Class of Medication

- ◯ Anticoagulants
- ◯ Antidepressants
- ◯ Antipsychotics
- ◯ Beta-blockers
- ◯ Diuretics
- ◯ (Other) Antihypertensives
- ◯ NSAIDs
- ◯ Narcotics
- ◯ Opioids
- ◯ Benzodiazepines
- ◯ (Other) Sedatives
- ◯

Dosage ______________________________

Frequency

- ◯ 1xday
- ◯ 2xday
- ◯ 3xday
- ◯ 4xday
- ◯ PRN

If PRN How often dow you take the medication? ______________________________

Name of Medication 13 ______________________________

Class of Medication

- ◯ Anticoagulants
- ◯ Antidepressants
- ◯ Antipsychotics
- ◯ Beta-blockers
- ◯ Diuretics
- ◯ (Other) Antihypertensives
- ◯ NSAIDs
- ◯ Narcotics
- ◯ Opioids
- ◯ Benzodiazepines
- ◯ (Other) Sedatives
- ◯

Dosage ______________________________

Frequency

- ◯ 1xday
- ◯ 2xday
- ◯ 3xday
- ◯ 4xday
- ◯ PRN

If PRN How often dow you take the medication? ______________________________

Name of Medication 14 ______________________________

Class of Medication

- ◯ Anticoagulants
- ◯ Antidepressants
- ◯ Antipsychotics
- ◯ Beta-blockers
- ◯ Diuretics
- ◯ (Other) Antihypertensives
- ◯ NSAIDs
- ◯ Narcotics
- ◯ Opioids
- ◯ Benzodiazepines
- ◯ (Other) Sedatives
- ◯

Dosage ______________________________

Frequency
- ◯ 1xday
- ◯ 2xday
- ◯ 3xday
- ◯ 4xday
- ◯ PRN

If PRN How often dow you take the medication? ________________________________

Name of Medication 15 ________________________________

Class of Medication
- ◯ Anticoagulants
- ◯ Antidepressants
- ◯ Antipsychotics
- ◯ Beta-blockers
- ◯ Diuretics
- ◯ (Other) Antihypertensives
- ◯ NSAIDs
- ◯ Narcotics
- ◯ Opioids
- ◯ Benzodiazepines
- ◯ (Other) Sedatives
- ◯

Dosage ________________________________

Frequency
- ◯ 1xday
- ◯ 2xday
- ◯ 3xday
- ◯ 4xday
- ◯ PRN

If PRN How often dow you take the medication? ________________________________

Name of Medication 16 ________________________________

Class of Medication
- ◯ Anticoagulants
- ◯ Antidepressants
- ◯ Antipsychotics
- ◯ Beta-blockers
- ◯ Diuretics
- ◯ (Other) Antihypertensives
- ◯ NSAIDs
- ◯ Narcotics
- ◯ Opioids
- ◯ Benzodiazepines
- ◯ (Other) Sedatives

Dosage ________________________________

Frequency
- ◯ 1xday
- ◯ 2xday
- ◯ 3xday
- ◯ 4xday
- ◯ PRN

If PRN How often dow you take the medication? ________________________________

Name of Medication 17 ________________________________

Class of Medication
- ◯ Anticoagulants
- ◯ Antidepressants
- ◯ Antipsychotics
- ◯ Beta-blockers
- ◯ Diuretics
- ◯ (Other) Antihypertensives
- ◯ NSAIDs
- ◯ Narcotics
- ◯ Opioids
- ◯ Benzodiazepines
- ◯ (Other) Sedatives

Dosage ______________________________

Frequency
- ◯ 1xday
- ◯ 2xday
- ◯ 3xday
- ◯ 4xday
- ◯ PRN

If PRN How often dow you take the medication? ______________________________

Name of Medication 18 ______________________________

Class of Medication
- ◯ Anticoagulants
- ◯ Antidepressants
- ◯ Antipsychotics
- ◯ Beta-blockers
- ◯ Diuretics
- ◯ (Other) Antihypertensives
- ◯ NSAIDs
- ◯ Narcotics
- ◯ Opioids
- ◯ Benzodiazepines
- ◯ (Other) Sedatives

Dosage ______________________________

Frequency
- ◯ 1xday
- ◯ 2xday
- ◯ 3xday
- ◯ 4xday
- ◯ PRN

If PRN How often dow you take the medication? ______________________________

Name of Medication 19 ______________________________

Class of Medication
- ◯ Anticoagulants
- ◯ Antidepressants
- ◯ Antipsychotics
- ◯ Beta-blockers
- ◯ Diuretics
- ◯ (Other) Antihypertensives
- ◯ NSAIDs
- ◯ Narcotics
- ◯ Opioids
- ◯ Benzodiazepines
- ◯ (Other) Sedatives

Dosage ______________________________

Frequency
- ◯ 1xday
- ◯ 2xday
- ◯ 3xday
- ◯ 4xday
- ◯ PRN

If PRN How often dow you take the medication? ______________________________

Name of Medication 20 ______________________________

Class of Medication

- ◯ Anticoagulants
- ◯ Antidepressants
- ◯ Antipsychotics
- ◯ Beta-blockers
- ◯ Diuretics
- ◯ (Other) Antihypertensives
- ◯ NSAIDs
- ◯ Narcotics
- ◯ Opioids
- ◯ Benzodiazepines
- ◯ (Other) Sedatives

Dosage ______________________________

Frequency

- ◯ 1xday
- ◯ 2xday
- ◯ 3xday
- ◯ 4xday
- ◯ PRN

If PRN How often dow you take the medication? ______________________________

Name of Medication 21 ______________________________

Class of Medication

- ◯ Anticoagulants
- ◯ Antidepressants
- ◯ Antipsychotics
- ◯ Beta-blockers
- ◯ Diuretics
- ◯ (Other) Antihypertensives
- ◯ NSAIDs
- ◯ Narcotics
- ◯ Opioids
- ◯ Benzodiazepines
- ◯ (Other) Sedatives

Dosage ______________________________

Frequency

- ◯ 1xday
- ◯ 2xday
- ◯ 3xday
- ◯ 4xday
- ◯ PRN

If PRN How often dow you take the medication? ______________________________

Name of Medication 22 ______________________________

Class of Medication

- ◯ Anticoagulants
- ◯ Antidepressants
- ◯ Antipsychotics
- ◯ Beta-blockers
- ◯ Diuretics
- ◯ (Other) Antihypertensives
- ◯ NSAIDs
- ◯ Narcotics
- ◯ Opioids
- ◯ Benzodiazepines
- ◯ (Other) Sedatives

Dosage ______________________________

Frequency
- ◯ 1xday
- ◯ 2xday
- ◯ 3xday
- ◯ 4xday
- ◯ PRN

If PRN How often dow you take the medication? ______________________

Name of Medication 23 ______________________

Class of Medication
- ◯ Anticoagulants
- ◯ Antidepressants
- ◯ Antipsychotics
- ◯ Beta-blockers
- ◯ Diuretics
- ◯ (Other) Antihypertensives
- ◯ NSAIDs
- ◯ Narcotics
- ◯ Opioids
- ◯ Benzodiazepines
- ◯ (Other) Sedatives

Dosage ______________________

Frequency
- ◯ 1xday
- ◯ 2xday
- ◯ 3xday
- ◯ 4xday
- ◯ PRN

If PRN How often dow you take the medication? ______________________

Name of Medication 24 ______________________

Class of Medication
- ◯ Anticoagulants
- ◯ Antidepressants
- ◯ Antipsychotics
- ◯ Beta-blockers
- ◯ Diuretics
- ◯ (Other) Antihypertensives
- ◯ NSAIDs
- ◯ Narcotics
- ◯ Opioids
- ◯ Benzodiazepines
- ◯ (Other) Sedatives

Dosage ______________________

Frequency
- ◯ 1xday
- ◯ 2xday
- ◯ 3xday
- ◯ 4xday
- ◯ PRN

If PRN How often dow you take the medication? ______________________

Name of Medication 25 ______________________

Class of Medication
- ◯ Anticoagulants
- ◯ Antidepressants
- ◯ Antipsychotics
- ◯ Beta-blockers
- ◯ Diuretics
- ◯ (Other) Antihypertensives
- ◯ NSAIDs
- ◯ Narcotics
- ◯ Opioids
- ◯ Benzodiazepines
- ◯ (Other) Sedatives

Dosage ________________________________

Frequency
- ◯ 1xday
- ◯ 2xday
- ◯ 3xday
- ◯ 4xday
- ◯ PRN

If PRN How often dow you take the medication? ________________________________

Name of Medication 26 ________________________________

Class of Medication
- ◯ Anticoagulants
- ◯ Antidepressants
- ◯ Antipsychotics
- ◯ Beta-blockers
- ◯ Diuretics
- ◯ (Other) Antihypertensives
- ◯ NSAIDs
- ◯ Narcotics
- ◯ Opioids
- ◯ Benzodiazepines
- ◯ (Other) Sedatives

Dosage ________________________________

Frequency
- ◯ 1xday
- ◯ 2xday
- ◯ 3xday
- ◯ 4xday
- ◯ PRN

If PRN How often dow you take the medication? ________________________________

Name of Medication 27 ________________________________

Class of Medication
- ◯ Anticoagulants
- ◯ Antidepressants
- ◯ Antipsychotics
- ◯ Beta-blockers
- ◯ Diuretics
- ◯ (Other) Antihypertensives
- ◯ NSAIDs
- ◯ Narcotics
- ◯ Opioids
- ◯ Benzodiazepines
- ◯ (Other) Sedatives

Dosage ________________________________

Frequency
- ◯ 1xday
- ◯ 2xday
- ◯ 3xday
- ◯ 4xday
- ◯ PRN

If PRN How often dow you take the medication? ________________________________

Name of Medication 28 ________________________________

Class of Medication
- ◯ Anticoagulants
- ◯ Antidepressants
- ◯ Antipsychotics
- ◯ Beta-blockers
- ◯ Diuretics
- ◯ (Other) Antihypertensives
- ◯ NSAIDs
- ◯ Narcotics
- ◯ Opioids
- ◯ Benzodiazepines
- ◯ (Other) Sedatives

Dosage ________________________________

Frequency
- ◯ 1xday
- ◯ 2xday
- ◯ 3xday
- ◯ 4xday
- ◯ PRN

If PRN How often dow you take the medication? ________________________________

Name of Medication 29 ________________________________

Class of Medication
- ◯ Anticoagulants
- ◯ Antidepressants
- ◯ Antipsychotics
- ◯ Beta-blockers
- ◯ Diuretics
- ◯ (Other) Antihypertensives
- ◯ NSAIDs
- ◯ Narcotics
- ◯ Opioids
- ◯ Benzodiazepines
- ◯ (Other) Sedatives

Dosage ________________________________

Frequency
- ◯ 1xday
- ◯ 2xday
- ◯ 3xday
- ◯ 4xday
- ◯ PRN

If PRN How often dow you take the medication? ________________________________

Name of Medication 30 ________________________________

Class of Medication
- ◯ Anticoagulants
- ◯ Antidepressants
- ◯ Antipsychotics
- ◯ Beta-blockers
- ◯ Diuretics
- ◯ (Other) Antihypertensives
- ◯ NSAIDs
- ◯ Narcotics
- ◯ Opioids
- ◯ Benzodiazepines
- ◯ (Other) Sedatives

Dosage ________________________________

Frequency

◯ 1xday
◯ 2xday
◯ 3xday

If PRN How often dow you take the medication?

DO NOT FORGET TO SELECT FORM STATUS AND SAVE FORM
