# Supplementary material for: Effect of a Home Health and Safety Intervention on Emergency Department Use in the Frail Elderly: A Prospective Observational Study
Source: West J Emerg Med. 2023 May 3;24(3):522–31. doi: 10.5811/westjem.58378 (PMC10284516; doi:10.5811/westjem.58378)
Supplement: Supplementary file 2 [file wjem-24-522-s002.docx]

**SUMMARY OF PRIDE TRAINING AND PROCEDURES.**

|  | HIPAA Privacy Training | HIPAA Security Training | Human Subject Protection Training | Didactic Module 1: Patient Assessment | Didactic Module 2: Environment Assessment | Didactic Module 3: Patient Education | Experiential Learning  Observation | IT/REDCap  Training | Orientation  (Right Seat/Left Seat) |
| --- | --- | --- | --- | --- | --- | --- | --- | --- | --- |
| Paramedics | X | X | X | X | X | X | X | X | X |
| Research associates | X | X | X |  |  |  |  | X | X |
| Nurses | X | X | X |  |  |  |  | X | X |
| Summer Interns | X | X | X |  |  |  |  | X | X |

- **TRAINING FOR ALL PRIDE EMPLOYEES**
- **STANDARD REGULATORY COURSES.** There were three standardized regulatory courses on the Yale learning management system that had to be completed by all of PRIDE’s employees. They included HIPAA privacy training, HIPAA security training, and human subject protection training. All regulatory courses were conducted online and had to be updated every two years in accordance with the Universities requirements.
- **IT TRAINING FOR IPAD USE AND REDCap®.** Provided to all employees, this course focused on the use of the iPad, familiarization with Research Electronic Data Capture (REDCap), and the methods for collecting data in the field.

**PARAMEDIC TRAINING.** The PRIDE trained paramedics to function as researchers and as community referral resources outside the traditional scope of the EMS and 9-1-1 systems. Those who were selected went through a rigorous set of knowledge checks, in-hospital training and additional instruction in the use of research tools before being beginning to work as PRIDE team members. Training was divided into four categories that include experiential learning, standard regulatory courses, didactic training, and program-specific IT training. Refresher training was conducted monthly as the program evolved.

- **EXPERIENTIAL LEARNING.**

Paramedics initially spent 24 hours of observation/shadowing time with case managers and care coordinators in the emergency department (ED) at the Yale-New Haven Hospital. The purpose of this training was to:

- Understand different categories for VNA services (nursing, PT, OT, speech) and the criteria needed to qualify for referrals
- Understand services offered by home hospice programs and criteria needed to qualify for referral
- Understand CMS/insurance coverage guidelines for skilled home services (i.e. covered services vs need for private pay)
- Observe professional interactions between care coordinators and VNA and families to facilitate outpatient care
- Observe ED course and patient/family experience for patients who have fallen at home
- **DIDACTIC TRAINING.** Conducted for the paramedics as part of their introduction to PRIDE, this training consisted of:

- Module 1- Geriatric Physiology and Patient Assessment: The objectives of this module were to: Recognize the conditions that contribute to falls, 2) Assess the patients’ fall risk, 3) Complete a thorough assessment of the study participant, 4) Identify, and if appropriate, quantify and document the risks.
- Module 2 - Environmental Assessment: The objectives of this module were to 1) Identify common trip hazards, 2) How to perform an environmental assessment of a residence, and 3) How to document findings.
- Module 3 - Patient Education: The objectives of this module were to 1) How to study participants on 1) How to reduce falls, 2) How daily activities increase resilience and strength, 3) What community resources are available, and 4) General health education.

**RESEARCH ASSOCIATES.**  PRIDE brought on researchers to work in the hospital’s emergency department (ED) to recruit study participants. In addition to the training required for all PRIDE employees, the Research Associates also had orientation created to introduce them to:

- Protocols within the YNHH ED
- Patient consent
- The use of EPIC® electronic medical record
- Infection control practices

**NURSE TRAINING.**  Training for the nurses (all of whom were already working for one of several collaborating home health care agencies) consisted of an orientation to the PRIDE Program, instructions on how to utilize PRIDE’s technology, specifically REDCap, Humanity® (ShiftPlanning) software, cloud-based workforce management and an employee scheduling program.

**STUDENT INTERNS/SUMMER HIRES –** Each summer as part of its community outreach PRIDE brought on student interns. Each student underwent an 8-hour orientation training which occurred during their first week of employment. Topics covered 1) an overview of the Program, 2) form usage tutorials, 3) University-required, on-line training on the HIPAA and human subjects research training, and 4) a review of recruitment and consenting procedures.

- **RIGHT SEAT, LEFT SEAT RIDE.** This was a process in which newly hired personnel would ride along and observe how experienced members of PRIDE performed in the field during home visits. Once the individual became familiar with the process the roles would be reversed where the experienced member observed and coached the new member on performing the home visit.

Initially this was done among the paramedics, but at the nurses’ request, it was implemented for them as well. PRIDE research associates used the same method to orient new team members to working in the Yale New Haven Hospital emergency department.
